# An Update in Knowledge of Pigs as the Source of Zoonotic Pathogens

**DOI:** 10.3390/ani13203281

**Published:** 2023-10-20

**Authors:** Agata Augustyniak, Małgorzata Pomorska-Mól

**Affiliations:** Department of Preclinical Sciences and Infectious Diseases, Poznan University of Life Sciences, Wolynska 35, 60-637 Poznan, Poland

**Keywords:** epidemiology, pigs, prevention, threat, zoonoses

## Abstract

**Simple Summary:**

The observed trend of increasing numbers of humans living on the Earth will result in increased demand for food, especially foods of high nutritional value, such as meat. This phenomenon will result in the growth of the livestock population. One of the most commonly consumed foods worldwide is pork. Therefore, it is reasonable to suspect that the pig world population, in the near future, will increase as well. Pigs are prone to infections/invasions with numerous viral, bacterial, and parasitic aetiological factors. Such infections are referred to as zoonotic infections. In view of the growing number of pigs headage, the possible risk of human infection of zoonotic origin seems to be increased. Therefore, the purpose of this paper is to investigate the most up-to-date data regarding the epidemiology of several of the most significant porcine zoonoses, with available preventive measures to control them.

**Abstract:**

The available data indicate that the human world population will constantly grow in the subsequent decades. This constant increase in the number of people on the Earth will lead to growth in food demand, especially in food of high nutritional value. Therefore, it is expected that the world livestock population will also increase. Such a phenomenon enhances the risk of transmitting pathogens to humans. As pig production is one of the most significant branches of the world’s livestock production, zoonoses of porcine origins seem to be of particular importance. Therefore, in this review, we aim to introduce the latest data concerning, among other things, epidemiology and available preventive measures to control the most significant porcine zoonoses of viral, bacterial, and parasitic origin.

## 1. Introduction

The One Health concept assumes that the health of humans, animals, plants, and the wider environment (including ecosystems) is closely related and interdependent. Although the One Health approach is not new, its significance has increased, especially recently [1]. This phenomenon results from numerous factors contributing to changes occurring in interactions between humans, animals, and the environment. Such changes include an increase in the international flow of people, animals, and animal-derived products, contributing to the faster spreading of diseases across the globe [1]. Other considered reasons are climate and land use changes, like deforestation and intensive farming practices [1]. Last but not least is the growth in the human population and its expansion to new geographical lands. The available estimations indicate that the human world population will constantly increase during proximate years and by 2050 reach approximately 10 billion [2]. Due to this, more people will live in the proximate neighbourhood of wild and domestic animals [1]. Such proximity to animals and their environments will offer more occasions for diseases to spill over from animals to humans [1]. These changes triggered the spread of known and emerging diseases of animal origin [1]. Human diseases derived from animals are called zoonoses. According to The World Organization for Animal Health (WOAH), nearly 60% and 75% of pathogens for human infections and emerging diseases are of animal origin [3]. These data underline the importance of the One Health concept. 

The continuous growth in the number of people on the Earth will result in higher food demand, particularly in foods of high nutritional value, such as meat [3]. Concerning FAO data, world meat production in 2020 was estimated at 337.2 million tonnes, and an additional 70% of proteins of animal origin will be necessary by 2050 to feed the human population. In 2022, world meat production was forecast to be 360 million tonnes, with a 1.2% increase compared to the previous year [4]. Regarding this fact, the world livestock population is also expected to increase [3]. Pig production is one of the most significant branches of the world’s livestock industry [5]. At the global level, within several years, pig meat production was second only to poultry production [5]. Moreover, the pig industry is a rapidly growing sector of the livestock world industry [6]. The observed expansion and intensification concerning the porcine industry led to substantial changes in classical pig husbandry practices, which resulted in the development of an environment that advantages the emergence and spread of infectious diseases [6]. Notably, pigs host a few pathogens that display pandemic or pre-pandemic properties capable of or have already caused a pandemic. Therefore, pigs, as reservoirs of zoonosis, should not be dismissed.

A large number of porcine infections can be transmitted to humans. Among porcine diseases, we can distinguish numerous zoonosis of viral (e.g., swine influenza, hepatitis E, Nipah, foot and mouth disease), bacterial (e.g., yersiniosis, erysipeloid, brucellosis, streptococosis, leptospirosis, *Staphylococcus aureus* infection), and parasitic origin (e.g., trichinellosis, *Taenia solium* invasions, cysticercosis), with various impacts on public health [7,8,9,10,11,12,13]. They can be transmitted in multiple ways, such as direct contact with an infected animal (Table 1). Due to this phenomenon, some porcine zoonoses are classified as occupationally related, as people who often have contact with pigs (e.g., farmers and veterinarians) are most likely to become infected. On the other hand, we have a group of porcine zoonoses that people can contract without any close contact with live animals, primarily due to consumption of infected pork; that puts individuals who are not related to pigs in any direct way at risk, as they often can not be aware of potential threats. Porcine zoonoses lead to human diseases with multiple symptoms in many world regions (Table 1). Some of them display very restricted geographical distribution; meanwhile, a large group is present worldwide (Table 1). Most of them can lead to severe clinical manifestations (Table 1). Moreover, some porcine zoonoses can represent a severe threat to public health, as their aetiological agents display pandemic or pre-pandemic properties. Generally, zoonoses of porcine origin are of worldwide distribution. They can be transmitted in multiple ways, generating risk for individuals who are occupationally related to pigs and those who do not have any direct contact with live animals, often leading to infections that can have severe clinical manifestations.

Due to the abundance of aetiological factors that can be transmitted between pigs and humans, it would be impossible to describe them all in one review paper. Therefore, we chose six diseases, of which pigs are considered one of the most important hosts, of various origins, transmission routes, world occurrence, severity of symptoms, and impact on public health to describe the diversity of porcine zoonoses. As the One Health approach aims, among others, to prevent outbreaks of zoonotic diseases in animals and people and improve food safety and security, we presented data concerning the current status, epidemiology, and prevention of chosen zoonoses [1].

## 2. Swine Influenza Virus

### 2.1. Aetiological Agent

Influenza A virus (IAV) is a single-strand, negative-sense RNA virus that belongs to the *Orthomyxoviridae* family [35]. The genome comprises 8 segments and encodes at least 12 proteins [36]. Based on the characteristics of two surface glycoproteins, hemagglutinin (HA) and neuraminidase (NA), IAV was divided into 19HA and 11NA subtypes [36,37]. IAVs are marked by fast evolution. This phenomenon occurs due to two mechanisms, namely antigenic drift and antigenic shift. The first one is quite often and results from the gradual accumulation of mutation of surface proteins that cause antigenic changes [38]. Antigenic shift (reassortment) occurs sporadically and is an effect of the shuffle and recombination of viral gene segments between different IAVs and leads to the development of new reassortants [38]. Numerous species of birds and mammals are susceptible to infection with IAV. However, only a restricted number of subtypes, such as H1 and H3, are established in mammals and circulate in, among others, pigs and humans [36]. Among IAVs of human and swine origin, there is a particular tendency for interspecies transmission [39]. Therefore, a bidirectional virus transmission between these hosts occurs [39].

IAV infection occurs due to the interaction between sialic acid (SA), which represents a receptor on the target cells, and viral HA [40]. Specificity of SA receptors, however, differs and is determined by the type of linkage between SA and subterminal galactose; human and swine IAVs preferentially bind to SA-a-2,6-terminal saccharides; meanwhile, avian IAV prefers SA-a-2,3-terminal saccharides [40,41]. Both SA receptor types were expressed in the porcine respiratory tract [41]. Moreover, the distribution of IAV receptors in pigs and humans is similar [40]. Therefore, pigs are susceptible to infection not only with swine IAVs but also avian and human IAVs, which puts them as a “mixing vessel” [40,41]. This phenomenon generates a risk of the development of new reassortants, also with pandemic potential [41,42]. Reassortment allows the development of novel antigenic and biological features [43]. Viral segments can interchange and recombine during simultaneous infection of a single host with at least two different IAVs, resulting in new viral reassortant [36]. The latest three IAV pandemics (1957, 1968, and 2009) were caused by reassortants of human and animal origin IAVs [43]. In 2009, the emergence of a new H1N1pdm09 IAV reassortant resulted in a worldwide human pandemic that caused at least 18,000 deaths globally [44]. H1N1pdm09 was distinct from other H1N1 viruses circulating at that time and was a product of reassortment between Eurasian avian-like swine H1N1 influenza virus (EA) and H3N2 North America triple reassortant (TR) [45]. 

### 2.2. Epidemiology in Pigs

The appearance of H1N1pdm09 strongly indicates the important role of pigs in new outbreaks [6,43]. The latest data confirm the above finding and highlight the importance of continuous global surveillance of swine influenza [43,46,47]. Swine influenza is spread worldwide. Currently, three main subtypes of IAV, namely, H1N1, H3N2, and H1N2, are co-spread in the global pig population [35,47]. However, the circulation of lineages belonging to those subtypes differs between particular continents (reviewed in Ma, 2020, and in Vincent et al., 2014) [14,36]. Of note, this includes H1N1pdm09, whose presence was already confirmed in the pigs’ population of all continents except for Antarctica [14]. Baudon et al. (2017) described the epidemiological features of swine influenza in pigs in their meta-analysis [48]. Concerning H1N1, H1N2, and H3N2, a high diversification in seroprevalence among the general pig population was documented. Nevertheless, increased values were noted in each continent, and the mean pig and herd seroprevalences ranged between 32.6 and 87.8%, as well as 29.3 and 100%, respectively. The lowest mean seroprevalences at the pig and herd level were estimated for Africa, and the highest for Latin America at the pigs level and for North America at the herd level. Generally, lower seroprevalence was documented in countries with low-to-medium GDP (gross domestic product). Moreover, some risk factors, including higher pigs density and the number of pigs per farm, are related to elevated prevalence. This may indicate that larger, industrialised farms are more endangered by swine influenza circulation. Notably, most studies from Europe, Latin, and North America were performed on pigs with influenza-like symptoms, which was linked with higher isolation rates [48]. The more recent data from Europe exhibited a high incidence of IAV in the European pig population, as 30.5% and 56.6% of examined samples and farms were IAV-positive, respectively [46]. The Chinese pig population also observed an increase in the annual isolation rate during 2011–2018 [43].

### 2.3. Epidemiology in Human

IAV can be easily transmitted between humans and pigs, probably due to a similar distribution pattern of IAV receptors between these two species [40,49]. Zoonotic transmission of swine influenza has been observed since 1918 [39]. Until 2009, the incidences of human-to-human transmission of swine-adapted IAV were relatively rare, with symptoms similar to those of seasonal flu [39]. The 2009 H1N1pdm09 was of porcine origin, which caused the most recent influenza pandemic, and it acquired the ability to spread quickly among humans, firstly across North America and subsequently around the world [50]. Collectively, 74 countries and territories have reported laboratory-confirmed cases [51]. Before the 2009 pandemic, the influenza A(H1N1) virus was never documented as a cause of human infection [51]. According to the WHO estimations, the number of cases during this pandemic ranges from several tens of millions to 200 million cases. By August 2010, approximately 18,500 laboratory-confirmed deaths had been notified; however, the number of overall deaths due to H1N1pdm09 can be much higher [51]. CDC estimated that 151,700–575,400 people died during the first year since the emergence of H1N1pdm09 due to infection with this pathogen [50]. At the global level, 80% of virus-related deaths were documented in people younger than 65 years old, and the pandemic primarily affected children and young and middle-aged adults [50]. Notably, the H1N1pdm09 pandemic had a less severe impact on the human world population than previous pandemics [50]. Currently, H1N1pdm09 circulates among humans as a seasonal flu virus, causing illness, hospitalisation, and deaths worldwide each year [50]. In addition to H1N1pdm09, cases of human infections with other IAVs of swine origins were also reported, but to a lesser extent [52]. Humans can contract an infection from pigs due to direct contact with sick animals, contact with contaminated surfaces, and inhaling the virus with droplets or dust [53]. Several studies indicate swine workers are at an increased risk of swine influenza zoonotic transmission [54,55,56]. Moreover, Gray et al. (2007) displayed that the increased risk of zoonotic influenza virus infections endangers non-swine-exposed spouses of swine workers [55].

### 2.4. Pandemic Potential

Henritzi et al. (2020) showed that pigs maintained in Europe constitute a reservoir for many IAV variants characterising zoonotic and presumably pre-pandemic potential [46]. Subtyping of positive specimens revealed the circulation of four main lineages (H1avN1av, H1huN2, H1pdmN1pdm, and H3N2) and seven minor reassortants. The simultaneous presence of several different IAV subtypes, including H1pdm, enabled reassortment and yield in genotypic diversification. In approximately 21% of surveyed isolates, internal genome segments of avian and H1pdm origin were identified. Phylogenetic analysis revealed the presence of 31 distinct genotypes of swine IAVs, including 16 that have not been reported previously in Europe. Moreover, surveyed swine strains of IAV exhibited large antigenic variations; it was documented that several H1pdm lineages antigenically different from seasonal human H1pdm lineages have simultaneously circulated in the pig population. Importantly, it has been proven that some currently circulating swine IAV isolates are partially or entirely resistant to MxA. This antiviral human protein is an essential factor that enables overcoming the species barrier in humans [46,57]. Furthermore, the experimental transmission of resistant strains among ferrets, a model of animal influenza transmissibility in humans, showed the capability to spread by direct or indirect contact [46].

Sun et al. (2020) demonstrated that Eurasian avian-like H1N1 is the primary IAV subtype that circulates in the Chinese pig population [43]. An annual increase in the mean virus isolation rate between 2011 and 2018 was observed, with significant growth since 2014. Lineage classification distinguished six genotypes (G1–G6) within Eurasian avian-like H1N1. One of them, G4, appeared in 2013 and, since 2016, has become the predominant genotype in at least 10 provinces. This newly emerged genotype was a reassortant of H1N1pdm09 and triple-reassortant strains and was antigenically distinct from remaining H1N1 Eurasian avian-like and pdm09 viruses. Similarly to H1N1pdm09, G4 was proven to preferentially bind to human-type receptors, which constitute an essential prerequisite for human cell infection and can effectively replicate in human bronchial and alveolar epithelial cells. Importantly, G4 displayed increased pathogenicity for ferrets, which served as an animal model for influenza infection in humans and was efficiently transmitted among these animals via aerosol. Serological surveillance conducted on specimens collected from individuals exposed to pigs revealed 10.4% seroprevalence among this group, indicating increased infectivity in humans. These findings raise concerns about the new pandemic virus, as G4 possesses all the features crucial for human adaptation [43]. Gu et al. (2022) observed high homology between two G4 swine isolates and contemporaneous human-origin EA H1N1 [58]. Moreover, these strains could effectively replicate in mice lungs without previous adaptation, and one bound preferentially to human-like receptors. Given the above research and the first confirmed human case of G4 infection, a possible threat to the public health of the G4 virus should not be ignored [58].

Yu et al. (2022) have described another swine IAV likely to have zoonotic potential [47]. Detected in China swine H3N2 virus with six internal genes originating from H1N1pdm09 and different antigenicity from contemporaneous human H3N2 IAV displayed higher affinity to human-type receptors and efficiently replicated not only in porcine but also in other mammalian cells, including humans. Furthermore, its ability to transmit between guinea pigs was also observed. Of note, this H3N2 can easily replicate and be spread among pigs. All these features may imply its potential zoonotic character and, together with numerous previous human cases of infection with other swine-derived H3N2 viruses, indicate the potential threat of this one [47].

### 2.5. Preventive Measures

All mentioned studies indicate that swine influenza still poses a great risk to public health as currently circulating strains display features predisposing to crossing the species barer from pigs to humans and sometimes exhibit pro-pandemic properties. Vaccination is the main tool to prevent IAV infection in humans and pigs. It is the best option for decreasing the opportunities for new reassortant development and the zoonotic spread of infection [59]. Currently, there are several commercially available products for the immunisation of pigs. They are based on whole inactivated virus (WIV) with adjuvant, live-attenuated influenza virus (LAIV), or alphavirus vectored RNA replicon particle (RP) [36,39]. Despite the large use of vaccines, effective control can be challenging due to the diversity and distribution of various IAV subtypes and genotypes [36]. Some commercially available vaccines, especially those based on WIV, can be inefficient in protecting against new IAV subtypes/clusters [39,60]. Commercially available WIVs used in pigs include two or more representatives of H1 and H3 cluster types [39]. A single WIV vaccine cannot fit all situations in one geographical region, which leads to an even greater problem on the world scale [14]. The data indicate that WIVs only partially protect against heterologous infections and viral shedding [39]. Therefore, WIV becomes considered a suboptimal method to control IAV [14]. However, in some countries, they are the only available kind of vaccine against swine influenza. LIAVs seem more efficient, as they provide good hetero-variant and partial heterosubtypic protection; however, they tend to reassort with viruses that endemically circulate [36]. Thus, regarding swine IAV, vaccine industries should focus on developing safe, effective, and rapidly altering antigenically matching emerging strain vaccines [60]. WIV and RP vaccines can be developed based on autogenous or custom farm-based strains [39]; such vaccines can be considered a more efficient alternative to commercial products as they will match field IAV. Importantly, the available data indicate that vaccines are widely used in the USA and, to a lesser extent, in Europe. However, due to various reasons, their usage in the rest of the world is not a common practice [48]. In Argentina, the lack of vaccination of pigs was considered one of the possible factors that contributed to the occurrence of new reassortants, which includes genes from H1N1pdm09 and human-like H1, and from H1N1pdm09 and wholly human H3N2 [14,61]. Therefore, educating farmers and veterinaries about the relevance of pig vaccination regarding swine influenza control seems essential. The results of several studies support the One Health perspective, in which pig industry workers should be included in annual seasonal influenza vaccination [54,56,62]. Such an approach can prevent the bidirectional transmission of IAV and decrease the potential for virus reassortment [56,62]. However, some of the detected in pig lineages, with presumably zoonotic potential, were antigenically distinct from human vaccine strains [46,47]. That may possibly enable the escape of humoral immunity already developed by the human population [46]. Vaccines are one of the most important approaches to controlling infectious diseases in pigs; however, many additional factors we are unaware of can influence their efficacy in this species [63]. Therefore, other preventive measures are also important. The CDC determined precautions to prevent the bidirectional spread of IAV, which include the following: frequent hand washing before and after exposure to pig or their environments, use of alcohol-based disinfectants, avoiding contact with pigs that look sick, avoiding contact when we are sick, using appropriate personal protective equipment such as gloves and a face mask, avoiding ingestion and putting anything to the month in the pigs’ area when there is a need to work with sick pigs, etc. Given the results from the study of Li et al. (2020), the possible risk to public health seems to be massive [64]. The mentioned research indicates a low awareness among people occupationally related to pigs (farmers, traders, and trade workers) about swine influenza being a zoonosis, as only 33.7% of respondents believed that people are susceptible to this infection. Furthermore, it was observed that dangerous practices that can increase the zoonotic risk of swine influenza, such as lack of personal protective equipment or influenza vaccination, seemed quite frequent in the evaluated population. Moreover, 12 out of 16 questioned trade workers admitted that they would enter the piggery to load animals. Among them, only six were undergoing disinfection procedures before entry. Over 80% of interviewees said they would still work with mild influenza-like symptoms [64]. It is partially consistent with the study of Ayim-Akonor et al. (2020), where a vast percentage of interviewees admitted that they do not use any masks or gloves and continue to work when they suffer from influenza-like symptoms [65]. Such practices put at risk not only these people but also possibly advantages increase the transmission of IAV from humans to pigs. Vincent et al. (2014) underline the need to improve swine influenza surveillance [14]. Collectively, swine influenza is especially important not only because it can lead to significant economic losses in the pig sector but also because it has a zoonotic potential, which represents a serious threat to public health worldwide [35,36]. Of note, concerning this disease, a bidirectional transmission occurs, and therefore, the One health approach is required to decrease morbidity and mortality in both hosts and ameliorate control strategies [39]. 

## 3. *Paslahepevirus balayani* (Hepatitis E Virus)

### 3.1. Aetiological Agent

*Paslahepevirus balayani* (hepatitis E virus—HEV) is a small, non-enveloped virus [66]. Viral genetic material is non-segmented, single-stranded RNA with positive polarity enclosed in an icosahedral capsid [20]. The genome length ranges between 6.6 and 7.3 kb [66]. With respect to the International Committee on the Taxonomy of Viruses, HEV is classified into the *Hepeviridae* family, *Orthohepevirinae* subfamily, which is further divided into four genera, namely *Paslahepevirus*, *Avihepevirus*, *Rocahepevirus*, and *Chirohepevirus*. *Paslahepevirus* consists of two species, *P. balayani* (previously *Orthohepevirus* A) and *Paslahepevirus alci* [66]. Eight different HEV genotypes (HEV1-HEV-8) have been distinguished so far, of which particular genotypes can infect various animal species, including humans. Next to the hepatitis A, B, C, and D viruses, HEV is the most frequent cause of viral hepatitis worldwide, and hepatitis E infection is considered an emerging zoonosis [67,68].

### 3.2. Epidemiology in Human

People are susceptible to infection with genotypes HEV-1-HEV-4, representing the main reservoir of genotypes HEV-1 and HEV-2 [20]. Infections with these two genotypes are mainly designated to developing countries; they are highly endemic in some Asian and African regions as well as in the Middle East and Mexico and may result in endemic and epidemic diseases [24,69,70]. The main route of infection with HEV-1 and HEV-2 is via contaminated faeces water [24,69]. Despite waterborne outbreaks, the transmission of infection due to direct contact, blood transfusion, or during pregnancy from mother to foetus is also possible [20]. Any confirmed cases concerning the transmission of HEV-1 or HEV-2 from animals to humans have not yet been documented, indicating that these genotypes do not display any zoonotic threat [20]. In contrast, infections with HEV-3 and HEV-4 are most often zoonotic and result from close contact with infected animals or ingesting HEV-contaminated food. Infections with these genotypes are mainly observed in developed countries as sporadic cases [69]. Of note, there is also one confirmed case of human infection with the HEV-7 genotype [71]. Such infection was documented in a patient from the Middle East, who regularly ingested milk and meat obtained from camels [71]. It was estimated that approximately 20 million cases of HEV infection emerge every year worldwide, resulting in an estimated 3.3 million symptomatic cases [72]. In 2015, hepatitis E led to 44,000 deaths, accounting for 3.3% of mortality due to viral hepatitis [72].

### 3.3. Epidemiology in Pigs

Initially, HEV infection was not linked to animals. It was thought to be a waterborne human infection that concerns only developing countries due to lack of drinking weather and poor hygiene. In 1997, the first pig HEV strain was isolated [73]. This strain displayed a similar genomic sequence to human HEV, suggesting that swine HEV can exhibit zoonotic potential. Moreover, HEV RNA was subsequently detected in pigs from developing and developed countries [67]. Currently, HEV is highly prevalent in the world pig population [74]. Anti-HEV antibodies and/or HEV genetic material were confirmed in numerous countries of almost all continents except for Antarctica [74]. Recent data indicate that, globally, nearly 60% of domestic pigs have undergone HEV infection. Moreover, approximately 13% of swine can be actively infected [74]. HEV prevalence rates vary between particular countries, regions, and farms [75]. Pigs represent the main reservoir of HEV-3 and HEV-4 [20]. HEV-3 causes the great majority of infections in this species. HEV-4 is less prevalent, and its distribution was observed in several European and Asian countries [74]. Infection in pigs with the remaining genotypes has not been observed to date. Therefore, pigs represent an important potential source of zoonotic HEV for humans.

### 3.4. Sources of Infection and Risk Groups

For a long time, HEV was incorrectly considered rare in industrialised countries [68]. It is known that HEV can also be highly prevalent in developed regions [68]. As mentioned, most infections in such areas are caused by genotypes HEV-3 and HEV-4, which display zoonotic origin. As the main animal reservoir of these genotypes is pigs, swine HEV has become an emerging public health concern, especially in such regions [75]. HEV-contaminated raw or undercooked pork products represent one of the primary sources of infections with HEV-3 and HEV-4 [75]. Numerous research from multiple countries confirmed the presence of HEV in various types of commercial pork food, mainly in livers and liver-derived products (Table 2). Especially alarming are reports concerning HEV in ready-to-eat products, which do not require heat treatment before consumption (Table 2). An estimation performed by Li et al. (2021) revealed that approximately 10% of pork products can be HEV-positive [74]. Another possibility for contracting the infection with HEV-3 or HEV-4 is contact with an infected pig. Therefore, individuals occupationally related to swine are in the group with the increased risk of HEV infection. For example, a recent study conducted in China showed that swine workers had higher HEV seroprevalence than the general population (47% vs. 26.1%) [76]. Moreover, it was observed that the infection threat for humans increased within the pork supply chain and was the highest in the slaughterhouses and pork markets [76]. It was hypothesised that this might result from higher exposure in such localisation to not only porcine faeces but also pig livers as well as other organs that are sites of HEV replication [76]. It is partially consistent with the study of Hoan et al. (2019), where individuals occupationally exposed to pigs displayed higher seroprevalence than unexposed controls [77]. The highest seropositivity was noted among slaughterhouse workers, followed by pig farmers and pork meat vendors [77]. A study performed in the United States indicated that swine veterinarians were 1.51 times more likely to be anti-HEV positive than controls [78]. A meta-analysis of 32 reports from 16 countries showed increased seroprevalence among pig workers [79]. On the other hand, some reports implied no significant association between seroprevalence and proximity to pig farms or swine workers with increased exposure [80,81]. It suggests that other factors, such as, for example, sanitation conditions and personal hygiene, influence HEV prevalence [75]. Xenotransplantation is another potential route of transmission of swine HEV to humans, as HEV presence was detected in triple genetically modified and non-modified animals bred for this purpose [24]. However, no HEV has been transmitted to non-human primates during preclinical trials [82]. Cases of such transmission via transplant organs from a human donor to a human recipient were observed [83].

### 3.5. Preventive Measures

The awareness of zoonotic HEV transmission is low [76]. Given the growing number of hepatitis E cases caused by zoonotic genotypes, pigs’ role in spreading this disease should not be diminished; therefore, control strategies should include pigs. As pork products represent one of the main sources of HEV-3 and HEV-4, appropriate treatment of such products that allows for virus inactivation should decrease potential risk. It was documented that cooking the meat at an internal temperature of 71 °C for 20 min efficiently deactivates HEV [21,22]. The surveillance of products that represent the HEV threat may also be a helpful tool [23,24]. Furthermore, proper safety measures should also be undertaken for different kinds of food possibly contaminated by porcine manure, such as shellfish, vegetables, and fruits [24]. Adequate measures should also be implemented to prevent HEV transmission via contact with infected pigs in people in the occupational risk group. This may include proper hand hygiene after contact with HEV reservoir animals or using personal protective equipment [25]. Denner (2019) has proposed strategies for eliminating HEV from pig herds bred for xenotransplantation and pork purposes [24]. This approach should involve selecting and breeding HEV-negative animals only, early weaning, Cesarian delivery, colostrum deprivation, embryo transfer, vaccination, and antiviral treatment [24]. Such strategies would certainly efficiently contribute to preventing HEV transmission from pigs to humans. However, they can be difficult to achieve [24]. Vaccines are one of the most efficient approaches for controlling viral diseases. There is one commercially available vaccine for humans (HEV 239 vaccine, Hecolin). However, it is not available anywhere else except in China. This vaccine is based on a protein encoded by ORF 2 of an HEV-1. It was proven to be highly efficient; it induces sustained levels of antibodies and confirms protection against hepatitis E for at least up to 4.5 years [20,84]. Moreover, it also efficiently protects humans against infection with the HEV-4 genotype [84]. Furthermore, as all HEV genotypes belong to the same serotype, it is suspected that this vaccine can protect against all HEV genotypes. However, this statement requires further studies [84]. This vaccine was also proven to be highly immunogenic for rabbits and conferred complete protection against infection not only with rabbit HEV but also with HEV-4 in this species [85]. These findings indicate that the HEV 239 vaccine can also be considered a candidate vaccine for managing HEV zoonotic sources [85]. However, there are no data concerning its efficiency in the pig model, and no vaccine is currently available for swine.

**Table 2:** Examples of countries in which HEV RNA was detected in pork products.

Country	Product	Reference
Belgium	Pork-liver pâté, raw-dried ham	[86]
Brazil	Pork-liver pâté	[87]
Canada	Liver, pork pâtés	[88]
China	Liver	[23]
France	Pork-liver sausage	[89]
Germany	Pork livers, spreadable liver sausages, liver pâté	[90]
Italy	Raw liver sausage, dry liver sausages	[91]
Netherlands	Raw pork sausages	[92]
Switzerland	Liver sausage, raw meat sausage	[93]
United Kingdom	Livers, pork sausages	[94]
United States	Liver, ground pork	[95]

## 4. Nipah Virus

### 4.1. Aetiological Agent and Pandemic Potential

Nipah virus (NiV) is an RNA virus considered one of the deadliest. It belongs to the *Mononegavirales* order, together with some other dangerous viruses, such as Hendra, Ebola, or Marburg [96]. Like the closely related Hendra virus, NiV uses conserved ephrin-B2 and ephrin-B3 as cellular receptors [97]. This phenomenon results in various hosts [97]. Frugivorous bats of the *Pteropus* genus are NiV natural reservoirs. Nevertheless, antibodies against NiV were detected in several species of domestic animals, of which pigs were the most frequently infected [98]. Although Nipah is an uncommon disease, it is an emerging zoonosis that causes a severe illness with high mortality and poses a high risk to public health worldwide [99]. Nipah is a stage III zoonotic disease [100]. This group includes diseases that can spill over to humans and lead to limited outbreaks of man-to-man transmission [100]. Zoonotic diseases are of particular public health concern as a source of human pandemics as they can cross the species barrier from animals to humans and be transmitted among people [100]. The ability to efficiently transmit between humans can establish these pathogens in the human population [100]. The pandemic potential of NiV is indicated by the following features: human susceptibility, the capacity of numerous NiV strains for man-to-man transmission, and high mutation rates [97,99]. Moreover, NiV is classified into category C of bioterrorist agents [101]. This group includes emerging pathogens that can be used for future mass dissemination due to their availability, ease of production and spread, and major health impact [101].

### 4.2. Epidemiology in Human

All main NiV human outbreaks that have occurred so far were restricted to the region of the geographical distribution of the *Pteropus* bats, including Malaysia, Singapore, India, Bangladesh, and the Philippines [31]. However, other countries, such as Cambodia, Ghana, Indonesia, Madagascar, and Thailand, are endangered by Nipah, as evidence of viral presence in the natural reservoir and other bat species has been demonstrated [102]. The first and most devastating Nipah outbreak was observed in 1998 among Malaysian pig farmers with the symptoms of severe febrile encephalitis; the newly emerged disease caused high mortality, as 105 out of 265 confirmed cases were lethal [103]. Primarily, the disease was linked to Japanese encephalitis, which is endemic in Malaysia; nevertheless, epidemiological characteristics between these two diseases were quite different [97,104]. Moreover, multiple strict measures introduced to control a new disease that occurred in pig farmers, which was thought to be Japanese encephalitis, have failed [103]. Contemporaneously, the emergence of a new pig disease marked by pronounced respiratory and nervous symptoms was observed in the same area; both diseases seemed to be closely related [103]. The discovery and isolation of NiV confirmed that this pathogen was responsible for infection in humans and pigs [103]. Subsequently, it was demonstrated that more than 90% of infected humans had contact with pigs; after that, it became evident that the emergence of the outbreak was linked to swine [97,105]. As the role of pigs in the spread of Nipah was initially underestimated, the movement of infected swine contributed to the spread of NiV infection to alternate areas [106]. It resulted in the emergence of new outbreaks in Malaysia and Singapore [106]. Direct contact with infected pigs and pig-derived products was primarily accountable for Malaysian outbreaks, as 92% of case patients admitted that they were handling pigs or were within 1 m of pigs and had contact with their urine or faeces [105,106]. The spread of NiV between pigs and humans probably occurs via the respiratory route of transmission [107]. By 2018, 643 Nipah cases from five countries were confirmed [107]. Of these, 380 cases were fatal, with a case fatality rate of 59% [107].

### 4.3. Epidemiology in Pigs

Pigs represent the intermediate host of NiV. The NiV spillover from bats to pigs resulted from the consumption of partially ingested or contaminated fruits by infected bats [108]. Convenient conditions, such as large numbers of pigs maintained in close proximity, subsequently enabled the wide spread of the virus [100]. Humans contracted the infection from pigs due to activities involving close contact with animals and their fluid or secretion, especially respiratory secretion and urine [105]. Interestingly, pigs were not linked with the subsequent outbreaks that occurred in humans after the Malaysian one, except for a few cases in Bangladesh [31,108]. Since 2001, Bangladesh has reported seasonal Nipah outbreaks yearly, except for 2002, 2006, and 2016, when any case was noted [102]. Contrary to Malaysia, infections in this country were mainly linked to the consumption of fresh date palm sap [109]. This variation may result from different husbandry model; in Bangladesh, animals are kept in a small group of livestock, which decrease the possibility of animal-to-human infection [31,100]. In contrast, large commercial farms in Malaysia maintain thousands of pigs in high density, which is advantageous for epidemic amplification [100].

### 4.4. Preventive Measures

Currently, pigs seem to not take part in the emergence of the new Nipah outbreaks; however, they played a significant role in the first and as-yet most devastating one. All major NiV outbreaks documented so far were restricted to Southwest Asia, an area with high human and pig density. This threatens the further spread of NiV to pigs and humans, and the possible adaptation of NiV to humans may result in a new, devastating pandemic [97,99]. This disease has no specific treatment; thus, prevention is essential. Biosecurity seems to be crucial in the preclude of the development of new outbreaks [31]. Regarding biosecurity, good practices in pig farming and the protection of crops against contamination by bats seem elementary [31]. Another important aspect is proper hygiene in the shape of frequent hand washing. In the outbreaks, staff working with pigs should wear personal protective equipment such as, among others, masks, gloves, protective goggles, and boots. Such equipment should be thoroughly cleaned and disinfected after each use [31]. The approaches for control of the disease should consider the development of an efficient vaccine not only for humans but also for pigs. An efficient vaccine for pigs could inhibit NiV spread and limit future outbreaks [97]. Efforts to develop efficient vaccines are in progress, and some research has given promising results in this area [31,97,110,111]. Despite that, no vaccine for either humans or pigs has been registered yet [31,97]. One vaccine (Equivac^®^ HeV; Zoetis, NJ, USA) is already registered in Australia and is used to protect horses and decrease zoonotic risk [112]. The experimental study showed that this vaccine was not similarly efficient in pigs [112]. Another practical approach to controlling Nipah seems to be surveillance for NiV in humans and animals that represents its reservoir, particularly in the areas where infection occurs. Such a solution would be early detection of new possible outbreaks that can emerge [107].

## 5. *Trichinella* spp.

### 5.1. Aetiological Agent

The nematode belonging to the *Trichinella* genus is a parasite of numerous animal species with a simple life cycle that entirely occurs within a single host, with no free-living stages [113]. The *Trichinella* genus consists of 10 different species and at least 3 distinct genotypes [113]. The two main clades in this genus were further distinguished depending on the presence or absence of the collagen capsule surrounding the muscle parasite. Notably, most taxa belonging to the *Trichinella* genus are classified as encapsulated. Meanwhile, the non-encapsulated clade consists only of three taxa—*T. pseudospiralis*, *T. papuae*, and *T. zimbabwensis* [99]. *Trichinella* spp. spread widely in all continents except Antarctica [114]. The distribution of each *Trichinella* spp. taxa belonging to both clades is mostly geographically restricted [115]. Among the encapsulated clade, each species’ distribution area differs from that of others belonging to the same clade, except for *T. spirallis*. This species is thought to be derived from the regions of Eastern Asia and subsequently was passively spread via the transport of pigs and pig products to other areas of the world, including Europe, North and South America, and New Zealand [115]. Representatives from different clades can coexist in the same area [115]. The transmission of this nematode occurs via ingesting live larvae contained in the meat [116]. Therefore, the carnivores and omnivores species are most vulnerable to this invasion [116]. Interestingly, species classified into the encapsulated clade can infect mammals only. Meanwhile, the representatives of the non-capsulated clade can parasite mammals, birds, and reptiles [114]. Regarding the circulation of this nematode among different species, two types of cycles, the synanthropic (domestic) and sylvatic (forest), occur [116]. 

### 5.2. Epidemiology in Human

*Trichinella* spp. are zoonotic parasites, which in humans lead to trichinellosis, a severe and sometimes fatal disease [16]. The species most frequently associated with human infections is *T. spiralis*; other species can also cause human disease [117,118]. To date, six species and two genotypes have been documented as a cause of human infections, including *T. spiralis*, *T. nativa*, *T. pseudospiralis*, *T. britovi*, *T. murrelli*, *T. papuae*, *T*. T6, and *T*. T9 [115,117,119,120]. *T. nelsoni* is a probable cause of human disease [115]. Trichinellosis occurs worldwide; cases of this disease in humans were confirmed in all continents except for Antarctica [121]. Until 2007, trichinellosis had been confirmed in 55 (27.8%) countries [121]. According to Zarlenga et al. (2020), the global incidence of human trichinellosis reflects the spread of this parasite among domestic and wild animals, eating customs, especially consumption of raw or undercooked meat and meat products, and the country’s socioeconomic development [113]. It is estimated that approximately 10,000 cases of trichinellosis occur yearly across the world, with a mortality rate close to 0.2% [121,122]. However, only a few countries have implemented an official recording system for infections, and, therefore, the epidemiological data can be fragmentary [121]. Moreover, the lack of pathognomonic clinical symptoms during this disease may lead to underestimating the number of human trichinelloses [113]. Over 11 million people are estimated to be chronically infected worldwide [118]. Notably, during the 21st century, we have observed a pronounced decrease in the number of infections noted in industrialised countries, such as Canada, China, the United States, and the members of the European Union [113]. For example, during the late 1940s, approximately 400 cases of trichinellosis were reported each year in the United States; meanwhile, only several cases are currently noted [122]. On the other hand, in other parts of the world, such as Argentina, Asia, and Eastern Europe, trichinellosis due to *T. spiralis* is considered emerging or re-emerging zoonosis [123]. The most recent data on human trichinellosis is presented as follows. Concerning ECDC data, in 2020, 9 out of 31 reporting countries collectively confirmed 117 cases of human trichinellosis. 88% of these cases were confirmed in three countries, namely Italy, Bulgaria, and Poland [124]. Moreover, Bulgaria and Italy noted the highest notification rate (cases per 100,000 population). Eighteen countries reported zero cases, four of which (Cyprus, Finland, Luxembourg, and Malta) have never reported any trichinellosis case before. The number of confirmed cases was higher than the previous years (97 cases in 2019 and 66 cases in 2018). However, it was lower compared to 2017 (168 confirmed cases) [124]. According to EFSA, in 2021, 77 cases of illness were detected in the EU [30]. Interestingly, compared to the previous year, the countries that accounted for 80% of all confirmed trichinellosis were Bulgaria, Croatia, Latvia, and Austria [30]. Overall, the trend for trichinellosis in the EU remained relatively stable within the last few years [30]. In the USA, between 2011 and 2015, on average, 16 cases were reported yearly [122]. Concerning South America, from 2005 to 2019, human trichinellosis was confirmed in Argentina and Chile only [125]. Between 2012 and 2018, 6662 suspected cases of this disease were noted in Argentina; meanwhile, in Chile, from 2005 to 2015, 258 cases were confirmed [125]. The study of Zhang et al. (2022) indicates that from 2009 to 2019, eight outbreaks with 479 cases of human trichinellosis and two deaths were confirmed in China [126]. Trichinellosis primarily affects adults, almost equally between men and women [118]. The main source of human infection is consuming raw or undercooked meat or meat products derived from animals that constitute *Trichinella* spp. reservoir, of which pork and wild game remain the main human trichinellosis source. According to the data obtained between 1986 and 2009, pork was the main source of infection during that period [118]. Currently, most trichinellosis outbreaks are due to the consumption of game [127]. However, infections resulting from the ingestion of pork still occur, especially in areas where backyard rearing of pigs for private, local purposes is common practice [127].

### 5.3. Epidemiology in Pigs

Pigs are one of the more than 150 animal species in which *Trichinella* infection was documented [128]. However, as mentioned previously, pork meat is one of the most important causes of human trichinellosis. Therefore, data regarding this infection in pigs are critical. Domestic and wild pigs represent the most significant reservoir of this nematode [113]. The most commonly observed taxa in pigs is *T. spiralis* [16]. This species is considered well-adapted to swine compared to remaining recognised members of the *Trichinella* genus; its larvae can last in the porcine muscle for at least 2 years [129,130]. Nevertheless, pig infections with other representatives of this genus, such as *T. britovi*, *T. nelsoni*, or *T. pseudospiralis*, are also possible [16,131]. However, the other species displayed lower larva survival and infectivity than *T. spiralis* [129,132]. *Trichinella* spp. in pigs is of worldwide distribution, as infections of pigs with this parasite were documented in numerous countries of all continents except for Antarctica [16,133]. Eslahi et al. (2022), based on 60 manuscripts originating from 32 countries from 2001 to 2021 and representing 65 pig populations, have estimated the pooled prevalence of *Trichinella* spp. in pigs worldwide as 2.02% [133]. The authors underline that due to the scarcity of data from some regions, the distribution and prevalence of *Trichinella* can be broader and higher than demonstrated via this meta-analysis. The study’s result revealed significant geographical differences in the distribution of *Trichinella*, as the assessed pooled prevalence in particular countries varied between 0.00% and 18.2%, reaching the highest values in Africa [133]. Significantly, in some locations, such as Greece or China, the positivity rates decreased over the years [133,134,135]. Observed differences in the estimated prevalence among particular countries are thought to be driven by the Human Development Index (HDI), pig husbandry system, and climate factors, as the highest values were reached in the low HDI countries, non-intense farming system and exotic, wet climate [133]. The most reliable data regarding *Trichinella* infection in pigs is derived from the EU, where all susceptible animals intended for the EU market should be tested for *Trichinella* larvae [136]. Due to this mandatory surveillance, the prevalence of pigs’ *Trichinella* infection in this area is well described, and we know that it occurs rather sporadically, mainly in animals not kept under controlled housing conditions. For example, in 2021, 0.0001% (120 out of 160 million) tested positive for *Trichinella* [30]. The highest number of infected pigs was confirmed in Romania (81), which was followed by Poland (19), Spain (13), Croatia (5), Finland (1), and France (1) [30]. Pigs, similar to other species, acquire infection via the oral route, mainly by the consumption of undercooked or raw meat (leftovers), by carcasses of infected animals (especially rodents), or via tail biting infected pigs [16,133].

### 5.4. Preventive Measures

Trichinellosis can be controlled on farms, at slaughter, and during the processing of meats [18]. The simplest way to avoid human trichinellosis of porcine origin seems to be to consume only adequately prepared pork meat. The International Commission on Trichinellosis (ICT) defined three processing methods to inactivate *Trichinella* before human consumption, including cooking, freezing, and irradiation [18]. Heat inactivation is considered very effective in preventing human infection [137]. ICT recommends cooking pork to an internal temperature of 71 °C [18]. Franssen et al. (2021) displayed that *Trichinella* muscle larvae lose their infectivity to mice after 12–12.5 min of exposure to a temperature of 60 °C or higher [137]. Freezing meat at the appropriate temperature and time efficiently inactivates this parasite [136]. Of note, two taxa, namely *T. nativa* and *T*. T6, are freeze-resistant [138]. As these taxa exhibit low infectivity to pigs, this control method can be implemented in pork [18]. However, where *T. nativa* and *T*. T6 are endemic, such infections should not be ignored [18]. At the slaughter level, prevention measures include testing meat [18]. In some countries, such as EU members, meat obtained from animals susceptible to *Trichinella*, intended for human consumption, must be mandatory testing for this parasite [136]. The magnetic stirrer is the most common and efficient technique in preventing human infection [139]. For example, compared to trichinelloscopy, this method effectively detects non-encapsulated larvae species and allows for the evaluation of multiple samples simultaneously [16,140]. However, despite its very high specificity, the artificial digestion method is marked by lower sensitivity regarding low-level infections [140]. Therefore, testing of carcasses indicates meat that is most likely to cause a clinical human disease if eaten unprocessed but does not prevent human exposure to *Trichinella* spp. [140]. In the areas where *Trichinella* infections are present in pigs, the potential risk of human exposure occurs despite the slaughter testing [19,140]. It is well documented that *Trichinella* spp. mainly circulates in pigs not kept under controlled management conditions [16,124]. Several consistent guidelines for *Trichinella* control, which included controlled management/controlled housing conditions, have been published over recent years [140]. Their main scopes include good feed manufacturing, effective rodent control, prevention of pigs’ contact with wildlife, farm hygiene, and pigs’ movement documentation [140]. Gamble (2022) considers that improvements in the pork industry that contributed to the decrease or elimination of pigs’ exposure to *Trichinella* spp. have an equal or even superior influence on the reduction of *Trichinella* spp. prevalence compared to long-standing testing programs [140]. A similar level of public health security between countries with implemented controlled management systems and countries where carcasses testing is performed seems to confirm such consideration [140]. The results of Franssen et al. (2018) indicate that controlling housing efficiently prevents trichinellosis, and testing does not impact food safety regarding this housing type [19]. Therefore, some authors indicate no need to test pork derived from pigs from controlled housing management [16,140]. Importantly, meat inspection and testing were efficient in preventing trichinellosis from pigs originating from non-controlled housing, as it was estimated that these methods could decrease the number of cases per year by 98.6% [19]. Pozio et al. (2014) suggest that all pigs from non-controlled management conditions, such as backyard and free-ranging pigs, should be mandatory testing for *Trichinella* spp. regardless of its intended use (market or private consumption) [16]. Approximately half of the world’s pork is currently produced under controlled management [127]. Nevertheless, an increase in the demand by consumers, especially from Europe and North America, for free-range pork is observed [127]. Regarding the fact that trichinellosis is strongly related to ingesting raw or undercooked meat, another important aspect of prevention seems to be the education of consumers and farmers [16,18]. It seems especially essential in populations in which, due to cultural conditions, dishes based on raw or undercooked meat are consumed [16]. The result of recent research conducted by Vieira-Pinto et al. (2021) demonstrated that 86% of responding hunters admitted that they intended to use meat for private purposes [141]. Of those, 93% conferred they sell part of this meat or homemade sausages, 80% without prior testing in the *Trichinella* direction [141]. The quoted data concern hunters and game, not farmers and pork, but exhibit a lack of awareness regarding the *Trichinella* threat among the increased risk group; therefore, education is pivotal. The decline in the incidence and health impact of trichinellosis seems to result from combined improvements in animal husbandry, the inspection of meat, education of consumers, and medical care [127]. Another promising approach for the control of trichinellosis seems to be veterinary vaccines [123]. The latest achievement in developing a veterinary vaccine against *T. spiralis* was carefully reviewed in two recent studies by Zhang et al. (2018) and Tang B. et al. (2022) [123,142]. Inventing an efficient vaccine could contribute to the improvement of animal health and prevent the transmission of infection to humans [123]. Several veterinary candidates for the *T. spirallis* vaccine have been proposed in recent decades [123,142]. The vaccine candidates included various antigens (excretory–secretory (ES) products, recombinant functional proteins, and some other antigens that participated in *T. spirallis* intracellular processes) and different types of vaccines (live-attenuated vaccines, natural antigen vaccines, recombinant protein vaccines, DNA vaccines, and synthesised epitope vaccines) [123,142]. To date, the development of efficient veterinary vaccines focuses on recombinant and DNA vaccines [123]. Despite the promising results of some studies, no effective vaccine is available yet [123].

## 6. Enteropathogenic *Yersinia*

### 6.1. Aetiological Agent

*Yersinia enterocolitica* (*Y. enterocolitica*) and *Yersinia pseudotuberculosis* (*Y. pseudotuberculosis*), together with *Yersinia pestis* (*Y. pestis*), are the three representatives of the *Yersinia* genus that are pathogenic for humans [7]. Meanwhile, *Y. pestis* causes a highly lethal and rapid disease named plague. *Y. enterocolitica* and *Y. pseudotuberculosis* are responsible for yersiniosis, a self-limiting infection [143]. *Y. enterocolitica* is, however, a primary cause of this disease, and infections due to *Y. pseudotuberculosis* are less frequent [144]. *Y. enterocolitica* is a Gram-negative, relatively anaerobic, psychrophilic enteropathogen that does not form spores [7]. Based on the biochemical features of this bacterium, six different biotypes were distinguished [27]. Among them, strains classified into 1b, 2, 3, 4, and 5 are considered pathogenic for animals and humans, of which 1b is thought to be highly virulent but remains a low-virulent biotype [27,145]. Biotype 1a was considered non-pathogenic due to the lack of the hallmark virulence markers [146]. Nevertheless, some 1a strains draw symptoms typical for pathogenic biovars. Thus, some authors concluded that 1a biovar could also have a pathogenic potential, perhaps as an opportunistic pathogen [147]. Furthermore, *Y. enterocolitica* was divided into over 70 serotypes [145]. This division is determined by the structure of the lipopolysaccharide O-antigen [145]. 

### 6.2. Epidemiology in Human

Infections with *Y. enterocolitica* are of worldwide distribution, as human infections with this pathogen were reported in various countries of most continents, including, among others, Africa (Nigeria), Asia (Bangladesh, China, Iran, Iraq, Japan), Australia (Australia, New Zealand), Europe (Germany, Finland, Poland, United Kingdom), North (Canada, the USA) and South America (Brazil, Chile) [144,148,149]. However, reliable surveillance data are available about only restricted regions of the world. According to ECDC, human yersiniosis is the third most frequently reported gastrointestinal infection in the EU [144]. In 2021, 6876 cases were documented in 28 countries, with the highest notification rates in Denmark, Finland, and Lithuania [144]. The overall notification rate has significantly increased compared to the previous year [144]. Nevertheless, the overall yersiniosis trend between 2017 and 2021 showed no statistically significant changes [30]. The highest notification rate was noted among the age group of 0–4 years [144]. Simultaneously, 115 infections with *Y. pseudotuberculosis* were reported by 11 members [144]. In the United States, it was estimated that *Y. enterocolitica* is responsible for approximately 117,000 illnesses, 640 hospitalisations, and 35 deaths yearly [150]. In China, this pathogen’s prevalence in children under 5 with diarrhoea was established as 0.59% [149]. In Bejing, the presence of this pathogen was confirmed in the diarrheal faeces of children and adults, and a higher prevalence was observed in the first-mentioned group [149]. In the subsequent study conducted in China, the isolation rate of *Y. enterocolitica* in children aged 6 months and 13 years with signs of acute diarrhoea was 0.80% [151]. Moreover, 85.71% of confirmed cases were noted in children younger than 5 years [151]. Despite the abundance of distinguished serotypes, only a few, such as O:3, O:5,27, O:8, and O:9, are mainly linked with human infections [152]. The bioserotype, which is most commonly associated with the disease in humans, is 4/O3. Moreover, 4/O:3, 1B/O:8, 2/O:9, and 2/O:5,27 predominated in human illness [27]. According to ECDC, in 2021, 4/O3 was responsible for 83.2% of infections reported in the EU and was followed by 2/O:9, which caused 15.3% of infections [144]. Moreover, 4/O:3 has become the most common cause of yersiniosis in North America, replacing the 1B/O:8 bioserotype, which was the predominant bioserotype in the United States until the 90s [153]. This bioserotype also seems dominant in other American countries, such as Brazil [154]. Duan et al. (2017) displayed that conversely, 3/O:3 is the most prevalent bioserotype in China, and other bioserotypes, such as 4/O:3 or 2/O:9, are rarely detected [149]. Interestingly, biotype 1A was found in 0.28% and 1.52% of diarrhoea cases in children and adults, respectively, and the majority of 1A isolates obtained from adults possessed the ystB gene, which suggests potential pathogenicity of these isolates [149]. Although the incidence of yersiniosis is carefully monitored in developed countries, there are no data concerning the incidence of this infection in Africa or the Middle East, probably due to a lack of adequate diagnostics in these areas [7]. The group, including children under the age of 5 years, older people, and individuals with decreased immunity, are at an increased risk of yersiniosis [7]. As yersiniosis is an enteric infection, transmission occurs via the oral route. Undercooked pork is considered the main source of *Y. enterocolitica* infection in humans [144]. Rosner et al. (2012) indicate that consuming raw minced pork was the leading risk factor for disease [155]. However, human infections can emerge due to cross-contamination of other food or food-related items while handling raw pork [144]. In the study of Merilahti-Palo et al. (1991), antibodies against *Y. enterocolitica* were detected more often in the sera collected from slaughterhouse workers, especially those who had handled swine throats and intestines (27%), in comparison to sera of healthy blood donors (10%) [156]. These results indicate that yersiniosis can represent an occupational risk to the employees slaughtering swine in the abattoirs [156].

### 6.3. Epidemiology in Pigs

*Y. enterocolitica* is widely distributed in the environment and was detected in numerous species of animals, including farm and wild ones [7]. Nevertheless, pigs represent their primary reservoir [7]. They are often asymptomatic carriers of this bacterium, as they do not develop any clinical signs of infection. This pathogen occupies the oral cavity, especially the tonsils and lymph nodes; its presence was also detected in the intestine and faeces (Table 3) [30]. Among pigs, *Y. enterocolitica* spread via the faecal–oral route [157]. Faecal shedding favours the spread of the infection within piggery; meanwhile, its carriage may contaminate carcasses [158]. Pigs represent the primary carriers and source of human enteropathogenic *Y. enterocolitica* [28]. In Europe, pigs most often carry strains belonging to bioserotype 4/O:3, followed by 2/O:9, 2/O:5, 2/O;27, which are less frequent (Table 3) [30]. Contrary to this, the most prevalent bioserotype detected in Asian pigs was 3:O/3 (Table 3). More detailed information concerning the detection of *Y. enterocolitica* in pigs is presented in Table 3. There is a lack of overall data concerning the worldwide prevalence of *Y. enterocolitica* in pigs. Such information is available only concerning the animals from the EU, where such surveillance is performed [30]. However, there are abundant studies from numerous countries in this direction (Table 3). The reservoirs of *Y. pseudotuberculosis* include wild and domestic animals; however, human infections are usually due to consuming contaminated vegetables [144]. Nevertheless, *Y. pseudotuberculosis* has also been detected in pig carcasses and pork, which implies the pig’s possible role in transmitting such infections [159]. Pigs are an important reservoir of *Y. pseudotuberculosis* bioserotype 2/O:3 [28]. The prevalence of this pathogen seems to be higher in organic production than in conventional production and on conventional farms with high production capacity [28].

### 6.4. Preventive Measures

The contamination of meat or meat products can occur at any stage of the food supply chain due to poor hygienic practices or inadequate handling and cooking processes [160]. *Y. enterocolitica* can be found in the non-ready-to-eat products [30]. Novoslavskij et al. (2013) suggest that the high contamination level of pig carcasses with enteropathogenic *Yersinia* species can be an important factor in the high incidence of human yersiniosis in Lithuania [28]. Laukkanen et al. (2009) have displayed that the high prevalence of pathogenic *Y. enterocolitica* bioserotype 4/O:3 in pigs may predispose to contamination of carcasses in slaughterhouses [161]. Contamination of carcasses with enteropathogenic *Yersinia* spp. most frequently originates from tonsils or faeces [159,161]. In the previously mentioned study, similar genotypes of pathogenic *Y. enterocolitica* 4/O:3 were isolated from pigs in farms, slaughterhouses, and from their carcasses and pluck sets, indicating that such contamination was due to direct contamination from the carrier pig [161]. In the case of pluck sets, cross-contamination with slaughterhouse equipment and the environment seem to play an important role as well [161], as these elements were proven to be often contaminated with the same genotype of *Y. enterocolitica* as pluck sets or carcasses [161]. Activities such as the removal of pluck sets or meat inspection represent a potential threat of pluck set contamination, as they involve handling elements that represent the contamination source and pluck sets [161]. Implementing rigorous hygiene procedures and educating workers can, thus, decrease cross-contamination [27]. Laukkanen et al. (2010) have displayed that bagging the pigs’ rectums in slaughterhouses was useful in reducing carcass contamination with *Y. enterocolitica* but not *Y. pseudotuberculosis* [159]. Moreover, when used alone, it is insufficient to prevent total carcass contamination, as high contamination rates can remain in the head and thorax area [159]. Other studies suggest that removing the head and tonsils can decrease contamination with *Yersinia* in abattoirs [162]. Drummond et al. (2012) suggest that serological testing could be an efficient approach for infection control at the slaughterhouse level [27]. In such a scenario, only uninfected animals would have contact during transport and in lairage, and they could be slaughtered together; meanwhile, positive animals would be separated. The reduction of this pathogen in farms should contribute to the decrease in the level of carcass contamination in abattoirs [161]. It was observed that the prevalence of *Y. enterocolitica* differs among particular farms, suggesting that some farm factors may influence it [161]. Nowadays, it is well known that numerous elements have an impact on the prevalence of enteropathogenic *Yersinia* spp. on pig farms; identification of such factors seems essential for developing and implementing efficient control measures that will allow for the minimalisation of enteropathogenic *Yersinia* spp. prevalence in farms and slaughterhouses [28]. Some previous studies indicated that the housing system could be an important factor influencing the prevalence of this pathogen in pig herds, as *Y. enterocolitica* was more prevalent in conventional and high-capacity farms in comparison to low-capacity farms and organic production [158,161,163]; however, the latest data seem to be in contrast to these findings [159]. Moreover, Laukkanen et al. (2009) have displayed that drinking from a nipple or the absence of coarse feed and bedding for slaughter pigs were associated with a high prevalence of enteropathogenic bioserotype 4/O:3; of note, the presence of the two last elements was more typical for organic farms with low animal density, contrary to nipple drinking, that was more common on large farms [158,161]. Novoslavskij et al. (2013) indicate that a low biosecurity level is associated with a high prevalence of *Y. enterocolitica* on pig farms [28]. The data concerning pest animals’ role in the pathogen’s spread in piggeries is inconsistent [28,161]. According to Virtanen et al. (2011), using municipal water and adequate commercial feed with some dietary supplements can be protective against bacterial carriage or shedding [158]. The risk factors for faecal shedding include tonsillar carriage, fasting pigs before transport to the slaughterhouses, and snout contact among pigs [158]. According to Skjerve et al. (1998), the steps that will allow reducing pigs carriage of *Y. enterocolitica* include farrow-to-finish production, an under-pressure ventilation system, the use of hygiene barriers for humans on herd entry, and clean straw bedding [29]. Drummond et al. (2012) underline the significance of basic biosecurity measures, such as disinfection and changing footwear before entering animal facilities or rodents and bird control [27]. Nesbakken et al. (2007), based on a specific pathogen-free (SPF) breeding pyramid, proposed an approach for producing *Y. enterocolitica*-free pigs [164]. Given the lack of medical prevention against yersiniosis, sanitary and hygienic conditions in slaughterhouses and public information campaigns seem to be essential prevention measures concerning this infection [26]. As inadequately thermally processed pork is the main source of human infections, public information campaigns seem more relevant [26]. To prevent human infections, pork meat should be consumed only when well-cooked, especially when served to children, representing a group of increased risk [30]. Prolonged cold storage of contaminated products increases the survival and growth of *Yersinia*. Proper, routine kitchen hygiene when handling contaminated food prevents cross-contamination [26,30]. Appropriate habits of the consumer that enable avoid infection include washing raw fruits and vegetables, consuming only pasteurised milk and dairy products, precise washing hands with the use of warm water and soap before and after touching any raw meat products, and cleaning all surfaces and equipment before and after food preparation [26]. The emergence of pathogenic strains of *Y. enterocolitica* resistant to antibiotics emphasises the need for surveillance of this pathogen [26,165]. In the future, the prevention of yersiniosis may also be achieved by vaccinations, as several studies on efficient vaccines have given promising results [143,166].

**Table 3:** Detection of enteropathogenic *Yersinia enterocolitica* in pigs.

Country	Positive Samples	Prevalence	Detected Bioserotypes	Comments	Reference
Brazil	Tonsils	10%	4/O:3		[167]
	Palate	10%			
	Head meat	5.9%			
Chile	Tonsils, rectal swabs	48%	4/O:3, 4/O:5	The predominant serotype was 4/O:3	[168]
China	Tonsillar swab	19.53%	3/O:3, 3/O:4, 2/O:9	The predominant serotype was 3/O:3	[169]
	Intestinal content	7.51%			
	Faeces	5.30%			
Estonia	Tonsils	89%	4/O:3	1% of samples were *Y. pseudotuberculosis*-positive	[170]
Finland	Tonsils	60%	4/O:3		[171]
	Intestinal content	26%			
Germany	Faeces	0.5%	4/O:3, O:9	The predominant bioserotype was 4/O:3	[172]
	Tonsils	38.4%			
	Ileocecal lymph node	3.8%			
	Carcass surface	0.3%			
Latvia	Tonsils	64%	4/O:3	5% of samples were *Y. pseudotuberculosis*-positive	[170]
	Tonsils	35%	4/O:3	10 samples were of Lithuanian origin	[173]
	Carcasses	13%			
Lithuania	Faeces	18%	4/O:3	Samples taken in slaughterhouses; 10% of faeces and 4% of carcass swabs were *Y. pseudotuberculosis*-positive	[28]
	Carcass swabs	25%			
	Faeces	22%	4/O:3	Samples taken at farms; 9% of samples were *Y. pseudotuberculosis*-positive	[28]
Malaysia	Nasal swab	17.6%	3/O:3		[174]
	Oral swab	15.2%			
	Rectal swab	12.7%			
Poland	Tonsillar swab	2.3%	4/O:3		[175]
	Rectal swab	64.9%			
Russia	Tonsils	34%	4/O:3	7% of samples were *Y. pseudotuberculosis-*positive	[170]
Sweden	Faecal samples	30.5%	4/O:3, 2/O:9	The predominant bioserotype was 4/O:3; faecal samples were pooled. Therefore, the prevalence relates to % of positive farms, not samples.	[176]
Switzerland	Tonsils	88%	4/O:3, 2/O:5,27, 2/O:9	The predominant bioserotype was 4/O:3	[177]
United Kingdom	Tonsils	44%	2/O:9, 2/O:5	The predominant bioserotype was 2/O:9; 18% of samples were *Y. pseudotuberculosi*-positive	[178]
United States	Faeces	13.10%	NE		[157]

## 7. *Eryspielothrix rhusiopathiae*

### 7.1. Aetiological Agent

*Erysipelothrix rhusiopathiae* (*E. rhusiopathiae*) is a facultatively anaerobic, gram-positive, non-spore-forming rod. It represents one out of ten species of the *Erysipelothrix* genus [179,180]. *Erysipelothrix* spp. is of worldwide distribution, and most isolated strains were classified as *E. rhusiopathiae* [181]. Historically, within *Erysipelotrix* isolates, based on heat-stable peptidoglycan antigens detected in a gel diffusion test, at least 28 serovars have been distinguished [34,179]. Specific serovars were assigned to particular species; for example, *E. rhusiopathiae* strains were classified into 17 serotypes [34,179]. Nevertheless, some studies indicate that such a division is not absolute, and particular serovars can be associated with more species than just one [34]. This bacterium was found to be a commensal or a pathogen for multiple species of vertebrate and invertebrate animals [182]. The terms erysipelas and erysipeloid describe this disease in animals and humans, respectively [34]. It is ubiquitous and can be found whenever nitrogenous substances decompose [33]. The presence of this pathogen was detected in water, soil, and food products, among others [33]. Moreover, it can survive long periods in the environment; in porcine faeces, the survival period ranges between 1 and 5 months [33]. The environment, however, is secondary in importance as a source of *E. rhusiopathiae* [33].

### 7.2. Epidemiology in Human

Human infections due to *E. rhusiopathiae* occur worldwide, documented in all continents except Antarctica [33]. Nevertheless, infections with this pathogen in humans are rare and appear as a sporadic disease [183]. A review of the literature published between 1950 and 2008 displayed that the disease was seldom reported during that time, as only approximately 50 articles on this subject were published [184]. It was more common in the past, and it is speculated that its incidence has decreased over the years due to some advances in the animal industry and sanitation [183,184]. Nevertheless, the study’s results by Rostamian et al. (2022) indicate that the number of confirmed cases has grown recently, and most cases were observed in developed and high-income countries [183]. Between 2000 and 2020, the highest number of identified human cases was noted in the United States, followed by France, Italy, Japan, and South Korea [183]. This phenomenon may result from better diagnostics in these countries and suggests that the number of infections in developing and undeveloped countries can be underestimated [183]. Infection in humans is occupationally related; it was documented that people with exposure to animals or their products in their workplace are more prone to infection [182,183]. Veterinarians, abattoir workers, butchers, fishermen, fish handlers, and homemakers are at the most significant risk of infection [182]. Rostamian et al. (2022) indicate that most patients, independently of their profession, admitted that they previously had contact with animals, which underlines the importance of such interactions in spreading the disease to humans [183]. The serovars were determined only in a few human cases of the *E. rhusiopathiae* infection and mainly belonged to serovars 1b and 2 [185]. Infection occurs due to skin injury with an infectious material or by contamination of some previous wounds; of note, most cases in human and animals are initiated by scratches or puncture injuries of the skin [8].

### 7.3. Epidemiology in Pigs

Initially, *E. rhusiopathiae* was established as a human pathogen [8]. Later, it was also identified as an animal pathogen, which could cause disease in multiple species, including pigs [8]. Pigs are considered one of the most significant reservoirs of *E. rhusiopathiae* [186]. The disease in pigs is of great prevalence and economic importance due to the substantial losses that can be caused in the porcine industry [8,33]. *E. rhusiopathiae* was isolated from pigs from almost all continents except Antarctica [186,187,188,189]. Therefore, swine erysipelas constitute a severe global problem [190]. Notably, despite improvements in management implemented over the years in the porcine industry, a recent increase in noted cases has been observed in various countries [191,192,193]. It is thought that the re-emergence of this infection may result from several factors, including changes in environmental conditions or restrictions on antibiotic usage [34]. Swine erysipelas is mainly linked to specific serotypes, namely 1a, 1b, and 2, as these serotypes are most frequently isolated from pigs with clinical signs of the disease worldwide [185,186]. Nevertheless, the remaining serotypes have also been detected in pigs with lower prevalence [186,194]. The disease is observed in growing pigs older than 3 months and adults [186]. Sick and infected pigs are the primary source of infection [190]. Asymptomatic pigs can carry this bacterium in their tonsils; depending on the research, *E. rhusiopathiae* has been found in 10.5% up to 98% of apparently healthy pigs [195,196]. The attenuation of animal immunity or an increase in the virulence of bacteria, resulting in endogenous infection, can lead to a disease outbreak [190]. Bacteria can be spread by carriers with faeces, urine, saliva, and nasal secretions, creating a threat of infection for humans and other animals and a risk of environmental contamination [8].

### 7.4. Preventive Measures

Although erysipeloid in humans is a relatively rare and self-limiting disease, in some cases, it can progress to bacteriemia and endocarditis with high mortality [183,190]. Due to unspecific symptoms and varying manifestations of the disease, diagnosis can be difficult, and many cases remain undiagnosed. Therefore, prophylaxis is essential, especially in groups with an increased infection risk. Control of the infection in the reservoir population can be challenging, if not impossible, due to the widespread distribution of this pathogen, its broad spectrum of hosts, and its ability to persist in the environments [8]. Education, safe work practices, and sensible precautions can decrease the possibility of human infection [8]. This pathogen mainly enters the host via injuries or wounds; thus, personal protective measures such as gloves, proper hygiene, especially frequent hand washing, and adequate treatment of injuries should be applied [8]. No vaccine is allowed for humans, and the immunisation of people does not seem reasonable, as clinical erysipeloid conveys little or no immunity [8]. *E. rhusiopathiae* is prone to commonly available disinfectants; regular cleaning and disinfection are, thus, necessary for limiting this organism’s spread around the work environment [8]. As pigs are considered one of the major reservoirs of the bacterium, efficient prevention among this species can contribute to better protection of human health. Control of the disease in pigs can be achieved by, among other things, proper husbandry and management, together with good sanitation [8]. Another approach to control the disease in pigs is vaccines, and erysipelas vaccines are commonly used in the porcine industry [34]. The current status of knowledge regarding the erysipelas vaccine was recently carefully reviewed by Opriessnig et al. (2020). All commercially available vaccines are based on serovar 1a or 2 isolates so far; serovar 1a is more commonly applied in attenuated vaccines; meanwhile, bacterins most often include serovar 2 [34]. Research trials concerning cross-protection among particular *Erysipelothrix* isolates are limited [34]. Some of them, however, showed that immunisation of pigs with vaccines based on serovars 2 strains also conferred protection against several other serovars [34,197,198]. Available erysipelas vaccines are considered efficient in preventing the disease; nevertheless, failures occur [34]. Various factors can be responsible for this. Commercially available vaccines may not be protective against field strains that represent different serotypes or Spa antigens than the vaccine strains [186]. Of note, available erysipelas vaccines are based on isolates obtained decades ago [34]. Little is also known about factors that confer protective immunity against *E. rhusiopathiae* infections. Therefore, research that will allow determining which of them, whether similar serovar, Spa type, some completely other protective antigens, or their combination, is crucial to confer cross-protection is desirable [34]. Moreover, it was documented that live vaccines against *E. rhusiopathiae* can result in side effects in the form of chronic erysipelas; live vaccine strains were responsible for approximately half of the cases of chronic arthritis-type swine erysipelas that have occurred during 11 years that have been involved in that study [199]. Given the growing number of reports concerning vaccine failures and the increase in erysipelas cases, novel, updated, and safer vaccine candidates are needed to improve protection [34]. Several new approaches for erysipelas vaccine development have been proposed and reached the preclinical stage. Such strategies include, among others, subunit vaccines based on SpaA antigen and attenuated ones as well [34].

## 8. Conclusions

This paper depicts the diversity of porcine zoonoses, especially in terms of their epidemiology, transmission routes, potential sources of infection, and their impact on public health. Most of them are spread worldwide, threatening humans across the globe. In the majority, they threaten individuals in direct contact with pigs or those who consume pork or pork derivative products. However, the disease can sometimes occur in humans unrelated to these animals. Therefore, a One Health approach, including humans and pigs, is required to prevent the spreading of such diseases in both species, as a decrease in the number of pig infections will help prevent human diseases.

Interestingly, despite the abovementioned variances between particular diseases, the types of used prevention measures seem similar. They are not the same in each of these diseases, as it is understandable that, for example, in the infections transmitted with pork, appropriate handling and treatment of meat is one of the most important prevention measures; meanwhile, in the case of infections that are not related to food it has no significance. However, many of the same groups of measures are implemented in the prevention of various diseases. They include, among others, appropriate hygiene, use of personal protective equipment, proper animal management, biosecurity, surveillance, and vaccinations, if they are available. This may suggest that such measures are universal, and it may be good to employ them routinely, independently of potential risk. More detailed activities in each category, i.e., choosing a disinfectant, should strictly match the pathogen and disease. The lack of awareness of the potential threat could be among the most important factors conducive to infections. Therefore, one of the essential elements of prevention, which often is unsung but can contribute to a decrease in the spread of infection in both pigs and humans, is the education of humans, especially those from risk groups, like individuals with direct pig contact or consumers of meat. Another issue that should not be diminished is that none of the abovementioned methods are 100% reliable. Therefore, to achieve the best results in this area, the implementation of more than one of the control measures should be considered. All things considered, complex proceedings based on a One Health approach are required to protect humans and decrease the number of zoonosis cases worldwide.

## Figures and Tables

**Table 1:** A brief characteristic of discussed zoonoses.

Disease	Swine Influenza	Trichinellosis	Hepatitis E	Yersiniosis	Nipah	Erysipelosis/Erysipeloid
Aetiological agent	Swine Influenza Virus (SIV)	*Trichinella* spp.	Hepatitis E virus genotypes HEV-3 and HEV-4	*Yersinia enterocolitica, Yersinia pseudotuberculosis*	Nipah virus (NiV)	*Erysipelothrix rhusiopathiae*
Distribution	Worldwide	Worldwide	Worldwide	Worldwide	Southwest Asia	Worldwide
Route of transmission	Contact with respiratory discharges or inhalation of exhalated aerosol by sick pig	Ingestion of raw or undercooked muscle tissue containing encysted larvae	Ingestion of pork; contact with infected pig	Ingestion of raw or undercooked pork; ingestion of contaminated vegetables or drinking water	Direct contact with infected pigs and pig-derived products	Injuries and wounds
Clinical manifestation—pigs	Fever, inactivity, decreased food intake, respiratory distress, coughing, sneezing, conjunctivitis, nasal discharge	Asymptomatic, intense muscle pain and reduced weight gains	Asymptomatic	Asymptomatic; diarrhoea in animals younger than 8 weeks	Asymptomatic, acute feverish illness, laboured breathing, trembling, twitching, and muscle spasms	Sudden death, septicaemia, erythema, cutaneous lesions, urticarial, diamond-skin lesions, arthritis, endocarditis
Clinical manifestation—humans	Sneezing, coughing, difficulty breathing, fever, lethargy, decreased appetite	Diarrhoea, abdominal pain, fever, myalgia, myocarditis, facial oedemas, encephalitis	Acute icteric hepatitis, malaise, fever, body aches, nausea, vomiting, dark-coloured urine, jaundice, liver failure, fatigue	Gastritis, enteritis, fever, stomach ache, diarrhoea (often bloody); complications include erythema nodosum, osteoarthritis, bacteraemia, purulent hepatitis, splenitis, nephritis, myocarditis, sepsis, endocarditis	Asymptomatic, acute respiratory infection (mild, severe), fatal encephalitis	Cutaneous infection, fever, joint aches, lymphadenitis, lymphadenopathy, arthritis, septicaemia, endocarditis, cerebral infection, intracranial abscess
Preventive measures	Vaccinations, proper hygiene and disinfection, personal protective equipment, surveillance, education of individuals occupationally related to pigs	Appropriate treatment of pork, testing of carcasses, maintaining of pig under controlled management conditions, education of consumers	Appropriate treatment of pork, surveillance of pork products, proper hygiene, vaccinations, education of consumers and individuals occupationally related to pigs	Proper hygiene, appropriate handling and treatment of pork, proper handling with carcasses, adequate pig management and biosecurity, education of consumers and individuals occupationally related to pigs, surveillance	Biosecurity, proper hygiene, personal protective equipment, surveillance, education of individuals occupationally related to pigs	Vaccinations, proper hygiene, adequate treatment of injuries, personal protective measures, biosecurity, education of individuals occupationally related to pigs
References	[3,14,15]	[3,16,17,18,19]	[20,21,22,23,24,25]	[3,7,26,27,28,29,30]	[31,32]	[8,33,34]

## Data Availability

Not applicable.
